# Microvascular contributions to white matter injury in Alzheimer’s disease

**DOI:** 10.18632/aging.204997

**Published:** 2023-08-21

**Authors:** Zsolt Bagi, Larry S. Sherman, Stephen A. Back

**Affiliations:** 1 Department of Physiology, Medical College of Georgia, Augusta University, Augusta, GA 30912, USA; 2 Division of Neuroscience, Oregon National Primate Research Center, Oregon Health and Science University, Portland, OR 97239, USA; 3 Department of Cell, Developmental and Cancer Biology, Oregon Health and Science University, Portland, OR 97239, USA; 4 Departments of Pediatrics, Oregon Health and Science University, Portland, OR 97239, USA; 5 Department of Neurology, Oregon Health and Science University, Portland, OR 97239, USA

**Keywords:** cerebrovascular, neuropathology, vasodilation, parenchymal, arteriole

Impairments in cognitive and executive function of presumed cerebral microvascular origin are important and recently recognized neuropathological manifestations of vascular contributions to cognitive impairment and dementia (VCID) [1, 2]. It has been long known that hypertensive cerebrovascular disease also involves a spectrum of subcortical small vessel diseases, such as arteriolosclerosis and lipohyalinosis of small penetrating arterioles, which contribute to progressive injury of periventricular, frontal and parietal white matter (WM). However, until recently, recognition of the role of WM injury during aging and the progression of Alzheimer disease and related dementias (AD/ADRD) was very limited. Despite growing interest in VCID and AD/ADRD, there have been few studies of mechanistic links between subcortical small vessel disease, WM injury and cognitive decline. Even though WM constitutes >80% of the human cerebral hemispheres, a PubMed search of AD and WM injury yielded only 381 articles (including reviews) vs. 193,303 articles for AD alone. Notably, 50% of diagnosed AD patients have mixed vascular and AD pathology. Hence, there is a critical need to explore connections between AD, WM injury, and cerebral small vessel disease to define mechanisms and diagnostic features of mixed vascular and AD neuropathological change (ADNC).

Studying WM continues to pose unique methodological and diagnostic challenges for aging research. WM is highly enriched in auto-fluorescent pigments that make rigorous immunohistochemical (IHC) approaches for mechanistic and diagnostic studies extremely challenging. Although astrocytes may degenerate in overt WM infarcts, the vulnerable glial subtypes in diffuse microvascular WM lesions remain essentially unknown. Admittedly, glial lineages are complex and relatively few specific markers are available. Furthermore, the expression of glial markers can change dramatically in regions of WM injury leading to nonspecific false positive staining [3]. In addition, WM is highly enriched in myelin lipids that obscure staining for key cellular markers and which make isolation of nuclei challenging for human single nucleus-RNAseq studies. These challenges are compounded by the lack of animal models of WM injury, particularly since rodent models have a paucity of WM with a blood supply strikingly different from humans. Despite these limitations, we recently demonstrated that high intensity LED arrays can be employed to photoquench autofluorescence in aging human tissue for fluorescence IHC and RNAscope studies [4]. This advance will allow rigorous quantitative fluorescent multi-labeling studies that can be harnessed to develop new diagnostic approaches to WM injury.

To provide rigorous access to human WM lesions, we recently developed a unique rapid autopsy brain procurement protocol using specimens donated by participants in the Adult Changes in Thought (ACT) study, a prospective, population-based study of aging and incident dementia among men and women in Seattle, Washington [5]. This protocol, developed by Dr. Dirk Keene in the Department of Laboratory Medicine and Pathology, University of Washington School of Medicine, deploys a unique neuroimaging-neuropathology pipeline where accelerated neuroimaging of human brains is achieved during rapid autopsy with a dedicated portable MRI scanner (Hyperfine Swoop). This unique neuroimaging resource developed by Dr. Christine MacDonald in the Department of Neurological Surgery, University of Washington School of Medicine, permits us to routinely image human brains less than 10 hours from death and immediately prior to brain collection. This protocol is uniquely adapted from the Allen Brain Institute to ensure accelerated hemibrain dissection into 4 mm coronal slabs that retain anatomical landmarks. Tissue slabs are alternately rapidly frozen in supercooled isopentane optimized for single cell and spatial omics or are paraformaldehyde-fixed to visualize novel markers, which are not detected in formalin-fixed tissue. The contralateral formalin-fixed hemisphere is subsequently imaged at 3 Tesla prior to detailed diagnostic studies to determine the extent of VCID or ADNC.

A unique opportunity afforded by this rapid autopsy brain procurement protocol is access to fresh tissue samples for direct analysis of human penetrating microvessels. We have developed novel protocols to preserve these vessels for a wide range of physiological and molecular studies performed within 24 hours after procurement. Although cerebral microvascular disease is a common manifestation of aging, prior human and murine vascular and morphological studies were limited mostly to the middle cerebral artery and pial microvessels, and findings were extrapolated to WM penetrating microvessels. Moreover, despite growing interest in WM injury and MRI-defined WM hyperintensities in VCID and AD, vascular studies mostly lacked histopathological, neuroimaging or mechanistic correlation.

We established the feasibility to study human penetrating WM arterioles in initial studies of aged human microvascular lesions with low ADNC [6]. We found an unexpected imbalance in vascular reactivity between pial and WM microvessels. Impaired vasodilator function of WM penetrating arterioles was associated with WM lesions in which oligodendrocyte progenitors failed to terminally differentiate to myelinating oligodendrocytes in astrocyte-enriched lesions [6]. Given that isolated microvascular brain injury is relatively uncommon, we recently asked how the combination of high ADNC and microvascular brain injury interact to influence vasodilator function of WM penetrating arterioles. Through a prospective study of serially collected human autopsy brains, we found that WM penetrating arterioles displayed the greatest impairment in vasodilator function in cases with both high vascular and AD neuropathological changes [7]. This selectively impaired vasodilator response of WM arterioles in cases with mixed vascular and AD pathologies was closely associated with abnormal WM microstructural integrity, as detected histopathologically by elevated reactive astrogliosis and by diffusion tensor imaging (DTI)-defined changes in ADC and FA values. Notably, we also previously found that that DTI measurements of human aging prefrontal WM were most significantly perturbed in cases with both cerebral microinfarcts and high ADNC [5]. Moreover, individuals with high ADNC (Aβ deposition and pathologic tau) and cerebrovascular disease develop cognitive impairment and memory deficits much earlier [2, 8]. It is therefore plausible that dysfunction of WM arterioles and high ADNC act synergistically to impair WM structural integrity in mixed vascular and AD pathologies.

**Figure 1:**
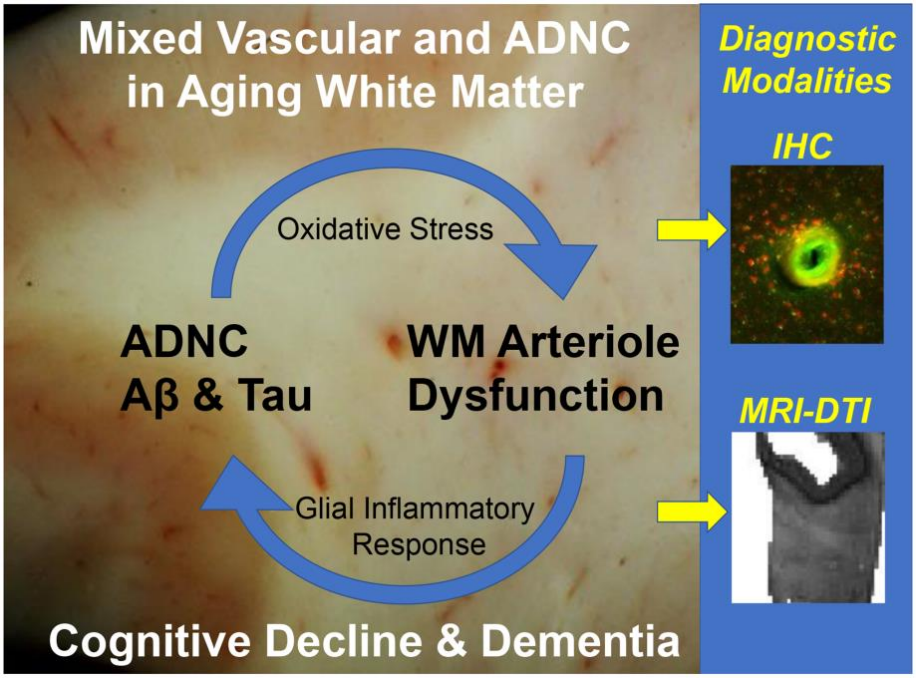
**A vicious cycle generating WM injury driven by mixed vascular and ADNC in the aging human white matter.** Diagnostic challenges for IHC and neuroimaging.

The molecular mechanisms that mediate enhanced dysfunction of WM parenchymal arterioles when vascular dysfunction and ADNC coincide remain elusive. Analysis of human WM lesions and WM parenchymal arterioles [5, 7], as well as preclinical models of AD with accumulation of toxic insoluble amyloid peptides, supports a role for excess production of reactive oxygen species. Notably, abnormal amyloid accumulation appears to trigger upregulation of NAD(P)H oxidases in the brain and increase their production of superoxide anion which can directly interfere with vasodilator signaling mechanism of cerebral microvessels. Importantly, impaired dilation of WM arterioles leads to subsequent hypoxemia and inflammation, which can exacerbate oxidative stress in association with axon and myelin damage and apparent failure of regeneration of myelinating oligodendrocytes [6]. It is therefore possible that the combination of pronounced microvascular dysfunction and ADNC sets up a vicious cycle that leads to disruption of WM microstructural integrity through mechanisms involving dysfunctional WM penetrating arterioles (Figure 1).

Taken together WM injury and WM hyperintensities of presumed microvascular origin are of growing interest to aging and VCID researchers. A growing body of evidence supports that microvascular brain injury is accompanied by oxidative stress, astrogliosis, changes in the extracellular matrix (including hyaluronan synthesis and catabolism), and quantitative MRI-defined abnormalities in prefrontal WM that involve disrupted integrity of axons and myelin. Mechanistic studies are needed to further define contributions of microvascular dysfunction to WM injury, particularly as it relates to the identities of the cell types and molecular signatures that could be harnessed to diagnostically discriminate among VCID, AD or mixed vascular/ADNC.
